# Theta synchronization between medial prefrontal cortex and cerebellum is associated with adaptive performance of associative learning behavior

**DOI:** 10.1038/srep20960

**Published:** 2016-02-16

**Authors:** Hao Chen, Yi-jie Wang, Li Yang, Jian-feng Sui, Zhi-an Hu, Bo Hu

**Affiliations:** 1 Department of Physiology, College of Basic Medical Sciences, Third Military Medical University, Chongqing 400038, P.R. China

## Abstract

Associative learning is thought to require coordinated activities among distributed brain regions. For example, to direct behavior appropriately, the medial prefrontal cortex (mPFC) must encode and maintain sensory information and then interact with the cerebellum during trace eyeblink conditioning (TEBC), a commonly-used associative learning model. However, the mechanisms by which these two distant areas interact remain elusive. By simultaneously recording local field potential (LFP) signals from the mPFC and the cerebellum in guinea pigs undergoing TEBC, we found that theta-frequency (5.0–12.0 Hz) oscillations in the mPFC and the cerebellum became strongly synchronized following presentation of auditory conditioned stimulus. Intriguingly, the conditioned eyeblink response (CR) with adaptive timing occurred preferentially in the trials where mPFC-cerebellum theta coherence was stronger. Moreover, both the mPFC-cerebellum theta coherence and the adaptive CR performance were impaired after the disruption of endogenous orexins in the cerebellum. Finally, association of the mPFC -cerebellum theta coherence with adaptive CR performance was time-limited occurring in the early stage of associative learning. These findings suggest that the mPFC and the cerebellum may act together to contribute to the adaptive performance of associative learning behavior by means of theta synchronization.

Associative learning of a motor response requires neural cooperation among distributed brain areas that perform sensory and motor processing^1^,^2^. One of the favored experimental models of associative learning is trace eyeblink conditioning (TEBC), which provides a good opportunity for studying cooperation among distributed brain regions^3^,^4^. This paradigm of conditioning experiment involves paired the presentations of a conditioned stimulus (CS; e.g., a tone) and a reinforcing unconditioned stimulus (US; e.g., a cutaneous electrical shock in the eyelid or a corneal airpuff), which are separated by a stimulus-free interval (trace interval)^1^. After receiving hundreds of paired CS-US presentations, the subject learns to respond to the CS, and to close the eyelid just before the US onset (conditioned eyeblink response, CR)^5^.

A large number of experiments have demonstrated that primary memory trace of the CR is located in the cerebellum and related brainstem circuitry^6^,^7^, although it has been shown that the cerebellum can also play a reinforcing role in the CR expression^8^. Intriguingly, to bridge a relatively long trace interval (e.g., 500 ms in duration) between the CS and US, the forebrain areas are recruited as support to facilitate the learning process^9^,^10^,^11^,^12^,^13^,^14^. Although many studies have implicated the forebrain areas, the neural mechanisms by which these structures perform their roles on TEBC have not been well-elucidated. One possibility lies in the coordinated activities between the forebrain and cerebellum, which are essentially involved in TEBC.

One such target forebrain region revealed to be activated during TEBC is the medial prefrontal cortex (mPFC). The mPFC has been revealed to connect with the cerebellum via the pontine nuclei in rodents^15^, rabbits^16^,^17^, non-human primates^18^ and the humans^19^. Moreover, multiple-unit recording studies have shown learning-related neuronal activity in the mPFC during TEBC^20^,^21^,^22^,^23^,^24^. Pharmacological or physical interventions that interfere with neuronal activity in the mPFC not only impair the expression of acquired trace CRs^3^,^4^,^25^,^26^, but also prevent the CR acquisition^12^,^13^,^14^.

The preceding reports have suggested that the mPFC might cooperate with the cerebellum during TEBC. It is believed that oscillation synchronization can provide a temporal window for optimal cooperation among distributed brain areas^27^. To demonstrate such cooperation during various behavior tasks, efforts have been made to measure oscillation synchronization between the mPFC and other areas. For example, theta oscillation synchronization has been revealed between the mPFC and the hippocampus during spatial memory^28^,^29^, the posterior association cortex during working memory^30^, and the visual cortex during visual short-term memory^31^. Whether the mPFC might utilize a similar neural mechanism to cooperate with the cerebellum is unclear.

To date, evidence has been accumulated that theta oscillation plays an important role in executing the TEBC task. For instance, theta oscillation contributes to synchronize neuronal activities at the distributed mPFC sites^32^, which in turn facilitates information transfer to the its target brain areas. Theta oscillations have also been revealed in the cerebellum^33^,^34^, which is associated with the adaptive performance of trace CRs^35^. At the single granule cell level, theta-band resonance may function as a reorganization of the output spike bursts on the millisecond time-scale, enhancing coherent spike emission from the granule cell layer in the cerebellum^36^,^37^.

Considering these studies implicating theta oscillation, we hypothesized that, during TEBC, oscillations in the theta range between the mPFC and the cerebellum might be synchronized. Moreover, synchronized theta-band oscillations might contribute to the memory performance of associative learning behavior. The current study tested these hypotheses by recording local field potential (LFP) from the mPFC and the cerebellum in guinea pigs during the TEBC task. In this study, theta-band (5.0–12.0 Hz) oscillation synchronization was observed between the mPFC and the cerebellum. Interestingly, the CR with adaptive timing occurred specifically when theta coherence was higher. Furthermore, both the mPFC-cerebellum theta coherence and the adaptive performance of CRs were impaired after functional inactivations of orexin-1 receptors in the cerebellum.

## Methods

### Subjects

The subjects in this experiment were 56 adult male albino Dunkin-Hartley guinea pigs weighing between 450 and 550 g (3–4 months old). Before the experiment and between the conditioning sessions, the animals were individually housed and had free access to food and water *ad libitum*. All experimental procedures were approved by the Animal Care Committee of Third Military Medical University and were performed in accordance with the principles outlined in the NIH Guide for the Care and Use of Laboratory Animals. All efforts were made to minimize the animals’ suffering and the use of the animals.

### Surgery

The surgical procedures had been recently described by Wang *et al.*^38^. Briefly, each animal was implanted with two single stainless steel electrodes (NO. 792300, A–M systems, Carlsberg, WA, USA). In 34 guinea pigs, one electrode (Covered diameter: 330.2 μm; Bare diameter: 254.0 μm) was implanted into the left intermediate cerebellum (beneath the lobules HVI and HVII, 2.5 mm lateral to midline, 5.0 mm ventral to skull surface, and 15.0–16.0 mm posterior to Bregma) according to an atlas of the guinea pig brain. The other electrode was implanted into the right mPFC (1.0 mm lateral, 2.0 mm ventral, and 1.5–2.5 mm anterior to Bregma). 4 anchoring stainless steel screws were implanted on surface of the skull. They were connected with each other to serve as reference (±5.0 mm lateral, 2.5 mm posterior to Bregma; ±5.0 mm lateral, 8.5 mm posterior to Bregma). This measurement was utilized to reduce the possibility that recorded neural oscillation in the cerebellum (or in the mPFC) only reflected activity at the reference site^39^. In addition, a stainless steel screw was anchored on the right cerebellum to serve as ground.

In another fourteen animals, an additional stainless steel guide cannula (No. 62001, Wrd, Shenzhen, China) was implanted into the left intermediate cerebellum, and a single stainless steel electrode (NO. 792300) was glued to the guide cannula. The tip of cannula was ~150 μm away from the recording site. This combination apparatus thus allowed us to perform drug injection in the site where was close to the extracellular LFP recording.

To evaluate LFP coherence between the mPFC and the hippocampus (HIP), 12 animals were implanted with two single stainless steel electrodes, with one electrode in the right mPFC and the other in the CA1 area of left HIP (4.0 mm lateral, 4.0 mm ventral, and 6.5–7.0 mm posterior to Bregma).

Finally, all animals were fitted with a headstage and a loop attached to the apex of the left upper eyelid. After surgery, all animals were allowed at least one week of recovery.

### Conditioning procedures

Following the postoperative recovery, all animals were adapted to the experimental environment for 2 days, 30 min per day. During daily adaptation sessions, the animals were restrained in a Plexiglas containing box located in a sound- and light-attenuating chamber with no stimuli given. The adapted animals then were randomly assigned to one of two experiments.

In this study, the CS was a tone (2 kHz, 85 dB, 350 ms, and 5 ms rise/fall time), which was paired with a corneal airpuff US (100 ms, 3.0 psi measured at the source) on the left side. The CS offset was separated by a 500-ms trace interval from the US onset. Daily conditioning training consisted of 5 blocks of 10 CS-US pairings trials for a total of 50 trials per session. The intertrial interval varied from 20 to 40 s with a mean value of 30 s, and the animals were trained for 10 consecutive days. As a control, the unpaired animals received an equal number of CSs and USs but never in the explicit temporal sequence that would induce associative learning. Accordingly, the inter-CS interval varied from 20–40 s with a mean value of 30 s. During conditioning training, a small hook was attached to the loop sutured into the left upper eyelid. This hook was connected to a high-resolution torque potentiometer (JZ-101, XH, Beijing, China). The analog output voltage of the potentiometer was used to quantify the eyelid position.

### Microinjections

Drug microinjections were administered 15 minutes before the beginning of conditioning training. We injected 1.6 μg (5.0 mmol/L, 1.0 μl) of selective orexin receptor-1 antagonist SB-334867 (Tocris, Bioscience, UK) through a stainless steel needle that extended 500 μm below the base of the guide cannula. The SB-334867 was dissolved in 1.0 μl, 0.9% saline and 5% DMSO (Sigma–Aldrich, St. Louis, MO, USA) prior to use. To validate the effectiveness of each cannula implantation, we injected 1.0 μg (8.8 mmol/L, 1.0 μl) of GABA_A_ receptor agonist muscimol (Sigma-Aldrich) into the left intermediate cerebellum on day 11. The muscimol was dissolved in 1.0 μl, 0.9% saline and 5% DMSO solution. The injections continued at a rate of 0.2μl/minute for 5 minutes. A constant injection rate was maintained using a syringe pump (CMA 402, CMA/Microdialysis, Solna, Sweden). The internal needle was kept in place for 10 minutes after each injection to allow the drug to diffuse from the needle tip. All the injected animals (*n* = 14) were allowed 24 hours to recover between test sessions.

### Recordings

The LFP signals were measured as the voltage difference between the single recording electrode and the reference screws placed above the skull. The LFP signals, eyelid movement mechanogram and markers of the applied stimuli were digitized at a sample rate of 10 kHz by a data acquisition system (Powerlab 8/35, ADInstruments, Australia) and were acquired using the software Chart (v. 7.3.7). The LFP signals were amplified 1000 times and band-pass filtered (0.5–30.0 Hz).

### Data analysis

#### Eyeblinks

For each training trial, we quantified the eyelid position for the baseline (1–350 ms before the CS onset), startle eyeblink response (SR; 1–120 ms after the CS onset), conditioned eyeblink response (CR; 121–850 ms after the CS onset), and unconditioned eyeblink response (UR; 1–500 ms after the US onset) periods. The SR period was determined on the basis of our previous quantification of guinea pig SR topography^40^. A significant eyeblink response was defined to exceed average baseline eyelid position by 1.0 mV (equaled to 0.25 mm movement) for minimal 15-ms duration. Any significant eyeblink response during the periods mentioned above was counted as a SR, a CR or an UR, respectively. To assess the potential correlation between the CR performance and LFP oscillation synchronization, we classified a CR as the adaptive or non-adaptive response, depending on whether the response reached the 200 ms period prior to the US onset^11^. Moreover, 5 parameters were used to evaluate the performance of non-associative eyeblink responses: (1) UR peak amplitude, (2) UR peak latency, (3) SR incidence, (4) SR peak latency, and (5) SR onset latency.

#### Local field potential

First, LFP signals from two recording electrodes were down-sampled at 1000 Hz using homemade software written in Matlab 2013a (MathWorks, Natick, MA, USA). For each training trial, a window size of 850 ms just before and after the CS onset was used. The LFP synchronization includes consistency of phase (phase coherence) and power correlation. In the present study, it was shown that theta coherence was not always high when signals recorded from the mPFC and the cerebellum showed strong theta oscillation (Fig. S1). Consequently, we evaluated the two measures separately as they may vary independently.

#### Phase coherence

For each conditioning trial, consistency of phase relationship was measured by calculating the instantaneous phases of two LFP signals through the Hilbert transform and subtracting the phases of the two signals from each other^41^. Phase coherence is always less than or equal to 1, taking a value of 1 only when the phase differences remain constant. By contrast, phase coherence will be zero if the phase differences are evenly dispersed among all epochs^41^,^42^. The phase differences were then plotted as a histogram, and the width of this plot at half of the peak was used as a measure of the consistency of phase relationship of two signals. If two LFP signals have a constant phase relationship, the phase difference histogram would show a narrow peak, regardless of the absolute phase differences.

#### Relative theta power

To evaluate the relative power of theta activity before (−850–−1 ms) and after (1–850 ms) the CS onset, Fast Fourier Transform (FFT) was performed on LFPs of these two periods with a resolution of 0.5 Hz, and was used to compute a power spectrum. To evaluate theta continuity, a sliding Hamming window continued to sample the incoming slow wave in 170 ms increments and re-calculated the ratio (510 ms previous data and 170 ms new data). Subsequently, a spectral ratio of the proportion of theta (5.0–12.0 Hz) to non-theta (0.5–4.5 Hz and 12.5–30.0 Hz) was calculated (relative theta power)^43^. Finally, linear theta power correlations between the mPFC and the cerebellum were calculated and averaged across the animals.

### Statistics

All data were expressed as the mean ± SEM unless otherwise noted. The statistical significance was determined by one-way or two-way ANOVA with repeated measures, independent or paired *t*-tests, as applicable, using the SPSS software for Windows package (v. 13.0). A minimum *p* value of <0.05 was an index of statistical significance for all tests.

### Histology

After completion of all the recording and behavioral experiments, the tips of recording electrodes in both the mPFC and in the cerebellum were marked by electrolytic lesions (3 mA for 20 s). Each animal was given a lethal dose of pentobarbital sodium (100 mg/kg) and perfused transcardially with saline followed by 4% paraformaldehyde (prepared in 0.1 M phosphate buffered saline, *pH* = 7.4). The brain was removed from the skull and fixed in formalin sucrose solution for 48 hours. Frozen coronal sections (30 μm) were collected from the recording and guide cannula sites. The slices were mounted on the gelatinized slides and stained with toluidine blue (Sigma-Aldrich). The locations of the recording electrodes and guide cannulae in the brain were histologically examined.

## Results

### Histology

To evaluate the relationship between neural activities in the mPFC and the cerebellum during TEBC, two single electrodes were separately implanted into the right mPFC and the left intermediate cerebellum (Fig. S2A). In this study, the location of each recording electrode was confirmed by histological analysis at the end of the experiments. In total, it was revealed that 34 animals had correct recording electrodes in both the mPFC and cerebellum (Fig. S2B). In the cerebellum, 16 electrode tips were located in deep cerebellar nuclei, and the remaining 17 electrodes were located in the area close to cerebellar cortex. In the mPFC, the electrode tips were located in the anterior cingulate cortex (ACC, *n* = 26) and the prelimbic area (PL, *n* = 7).

### Behavioral performance

**Figure 1:**
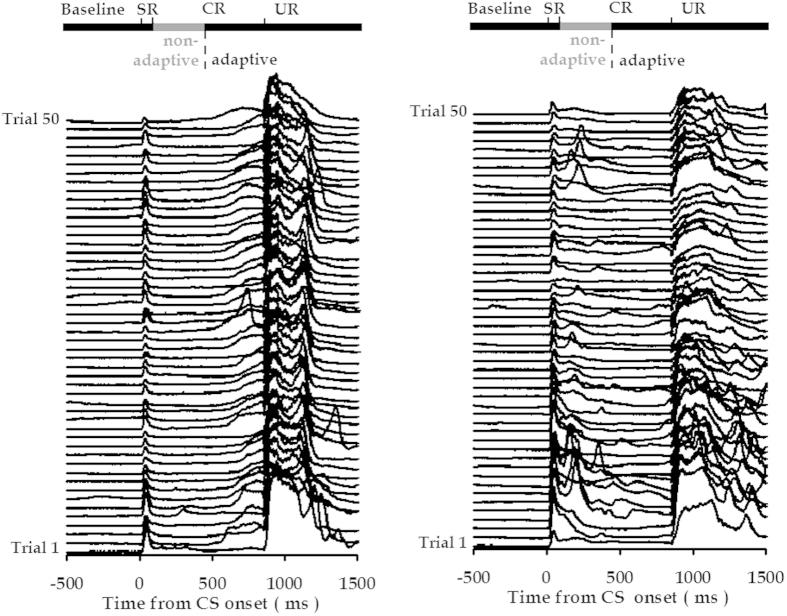
Classification of trace CRs into adaptive and non-adaptive responses. Individual examples of eyelid movement stack plots showing adaptive and non-adaptive CRs. The first trial for each 50-trial training session is at the bottom of the stack plot and each eyeblink trace represents one training trial. Left panel: A representative animal consistently showed adaptive CRs with peak latency close to the US onset. As shown, the adaptive CR persisted until the UR was exhibited. Right panel: A representative animal emitted non- adaptive CRs with an earlier peak. In particular, these responses returned to baseline before the 200-ms pre-US period.

In this study, we observed two types of CRs across TEBC training. As illustrated in Fig. 1, one type of CR was defined as adaptive response, because it occurred in 200-ms time area before US onset. On average, these responses started at [628 ± 104] ms (*n* = 3031, mean ± S.D.) after the CS onset, and persisted until the URs were exhibited. The other type of CR was defined as the non-adaptive response (Fig. 1, right panel). The statistical analysis showed that the non-adaptive CRs stopped at [301 ± 120] ms (*n* = 1919, mean ± S.D.) prior to US onset, which did not occur in the 200-ms pre-US area. Based on these observations, we classified the CS-US paired animals (*n* = 23) into two subgroups: adaptive *vs.* non-adaptive learners. The former subgroup of animals (*n* = 11) consistently exhibited adaptive CRs across conditioning training. In contrast, the latter subgroup of animals (*n* = 12) showed clear non-adaptive CRs.

**Figure 2:**
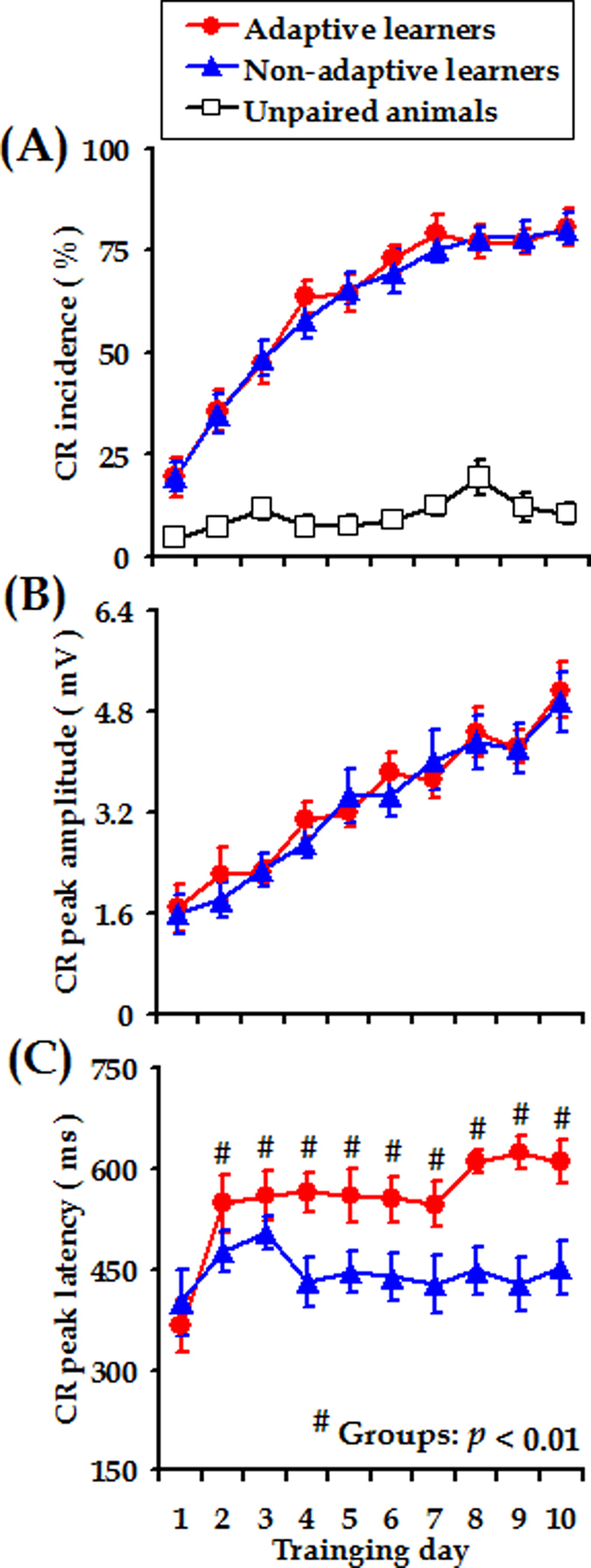
Acquisition of trace CRs in guinea pigs. (**A**) Incidence, (**B**) Magnitude and (**C**) Peak latency of trace CRs measured from the adaptive learners (red circles), non-adaptive learners (blue triangles), and unpaired animals (open squares), respectively. Data are given as the mean ± SEM. Symbols are the same for (**A**–**C**).

Figure 2 depicts the incidence and topography of CRs across 10 training days. As can be seen, the unpaired animals did not learn the CS-US association as measured by the CR incidences across conditioning training (*n* = 11, *F*_[9, 90]_ = 3.384, *p* = 0.093, mean incidence across ten days: [10.2 ± 0.9] %, Fig. 2A). Compared to the animals receiving pesudorandomized presentations of the CSs and the USs, the animals receiving paired CS-US presentations exhibited obvious increases in both the CR incidence and CR magnitude as conditioning progressed, indicating the improvement in their associative learning performance. ANOVAs with Day of training as a within-subject factor revealed significant effects of training day on both the CR incidence (adaptive learners: *F*_[9, 90]_ = 38.380, *p* < 0.001, *n* = 11; non-adaptive learners: *F*_[9, 99]_ = 36.420, *p* < 0.001, *n* = 12, Fig. 2A) and CR peak amplitude (adaptive learners: *F*_[9, 90]_ = 14.955, *p* < 0.001, non-adaptive learners: *F*_[9, 99]_ = 14.616, *p* < 0.001, Fig. 2B). Moreover, across 10 days of training, the non-adaptive learners exhibited similar CR incidence and magnitude to the adaptive learners (Incidence: *F*_[1, 21]_ = 0.050, *p* = 0.826; Magnitude: *F*_[1, 21]_ = 0.107, *p* = 0.747, Fig. 2A,B). However, CR peak latency in the adaptive learners was significantly later than that of CRs in the non-adaptive learners (Days 2–10, *F*_[1, 21]_ = 11.206, *p* = 0.003, interaction between Day of training and Group, *p* > 0.05, Fig. 2C). This result is consistent with our aforementioned expectation that the adaptive learners would exhibit maximum eyelid closure in response to the US onset, whereas the non-adaptive animals would not.

It should also be noted that there were no obvious differences in the topography of UR to the airpuff US and of SR to the auditory CS (Table S1) between the adaptive and the non-adaptive learners. Our results thus indicated that the two subgroups of animals were likely to have similar general motor responses or somatic sensation related to TEBC.

### Coherent theta-band (5.0–12.0 Hz) oscillations between mPFC and cerebellum

**Figure 3:**
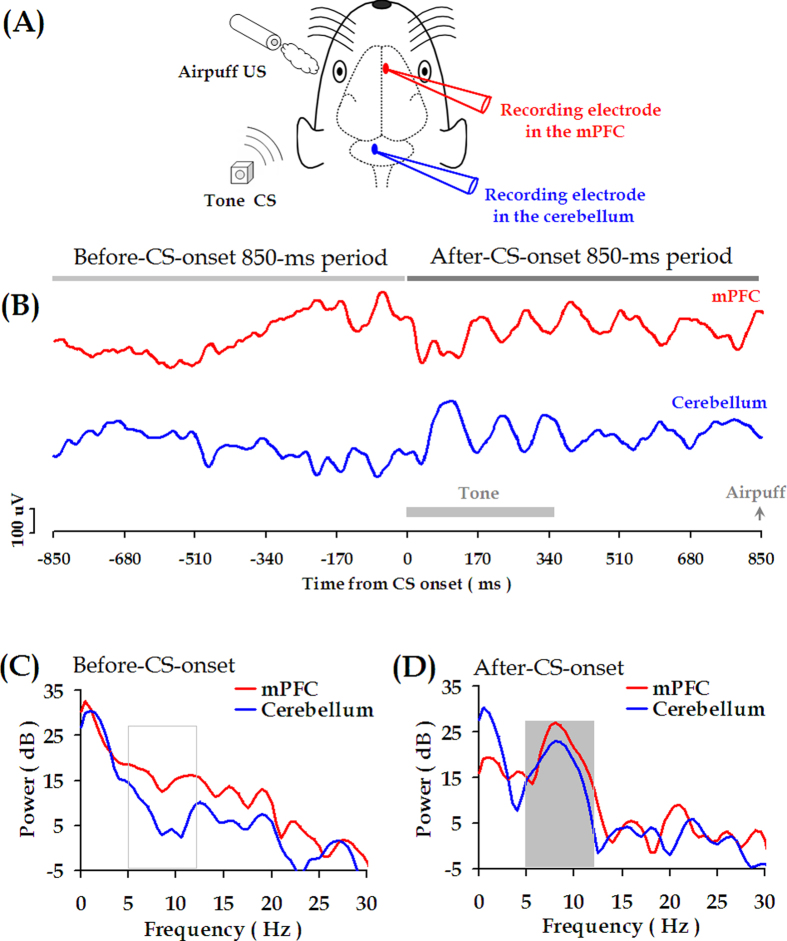
Theta-band oscillations (5.0–12.0 Hz) in mPFC and cerebellum. (**A**) Schematic diagram of simultaneous local field potential (LFP) recording in the mPFC and the cerebellum during TEBC. (**B**) In a representative CS-US trial, the LFP signals are recorded from the mPFC (red trace) and the cerebellum (blue trace) during 850-ms periods before and after CS onset, respectively. (**C**,**D**) Fast Fourier transform (FFT) runs on the mPFC and cerebellar LFP signals during the 850-ms periods before and after CS onset, as shown in (**B**).

In this study, LFPs signals in the mPFC and the cerebellum were simultaneously recorded during daily conditioning training (Fig. 3A). An example of the recorded raw LFP signals was illustrated in Fig. 3B. As we reported previously^35^,^44^, the auditory CS could evoke obvious rhythmic oscillations in both the mPFC and the cerebellum. The frequency of oscillation was 5.0–12.0 Hz, as shown in power spectrum computed from the raw LFP traces (Fig. 3D). Also, these theta-band oscillations span the periods of tone stimulus and trace interval (Fig. 3B).

**Figure 4:**
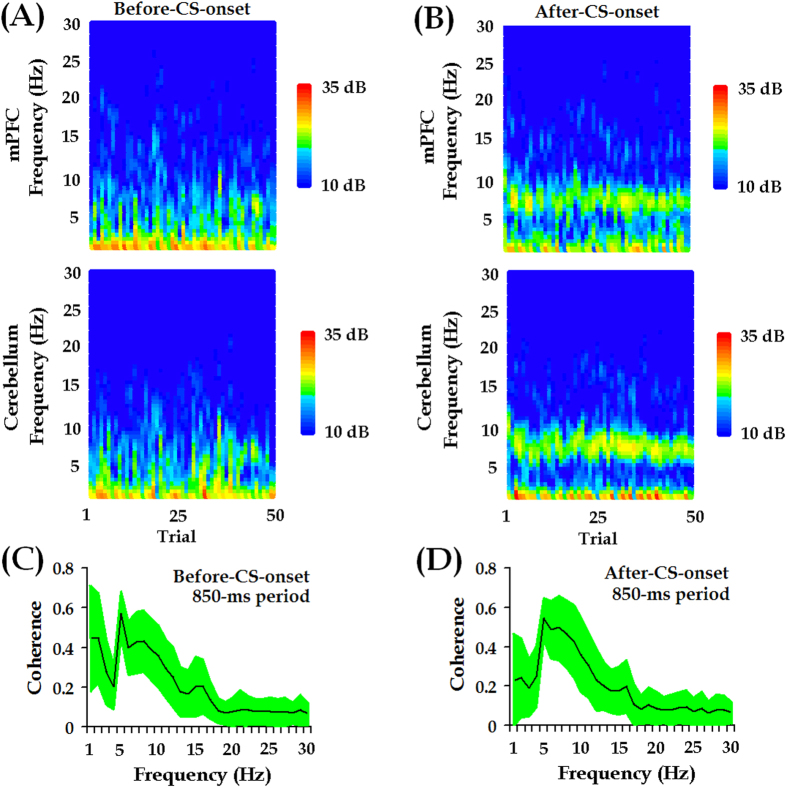
Coherent theta-band (5.0–12.0 Hz) oscillations between mPFC and cerebellum. Across a conditioning session, theta-band oscillations consistently occur in 850-ms periods before (**A**) and after (**B**) the CS onset, whereas theta-band activities are more evident in the latter period. (**C**,**D**) In the theta-frequency (5.0–12.0 Hz) range, the LFP signals recorded from the mPFC are highly coherent with the LFP signals from the cerebellum. Green-shaded areas indicate 95% confidence intervals.

As noted by Sarnthein *et al.*^30^, our term ‘synchronization’ is used for any phase-locked correlated LFP signals, not only ones with zero-degree phase lag. Therefore, an enhanced coherence might signify increased synchronization between two LFP signals. Intriguingly, after the CS onset, LFPs from the mPFC were phase-locked to LFPs from the cerebellum (Fig. 4), indicating that LFP signals from the two regions might be highly coherent. Indeed, across 50 conditioning trials, coherent mPFC-cerebellum oscillations were observed after CS onset, which predominantly peaked in the theta-frequency (5.0–12.0 Hz) range (Fig. 4D).

### mPFC-cerebellum theta-band synchrony remained relatively stable across training, but was stronger during the performance of adaptive CRs

We subsequently investigated whether theta synchronization changed over the course of conditioning training. To this end, the temporal relationship between the two LFP signals was examined session by session. Notably, in this study, special attention was paid to theta-band synchrony during the post-CS-onset period for three reasons. First, theta-band oscillations were most prominent in the post-CS-onset period rather than in the baseline period (Figs 3B and 4). Second, theta-band synchronization became higher following the CS presentations (Fig. S3 A1,A2). Third, the CRs were limited to occur in the period after CS onset, which allowed us to associate theta synchronization with behavioral performance. As a consequence, we measured LFP coherence in the 850-ms post-CS-onset period.

**Figure 5:**
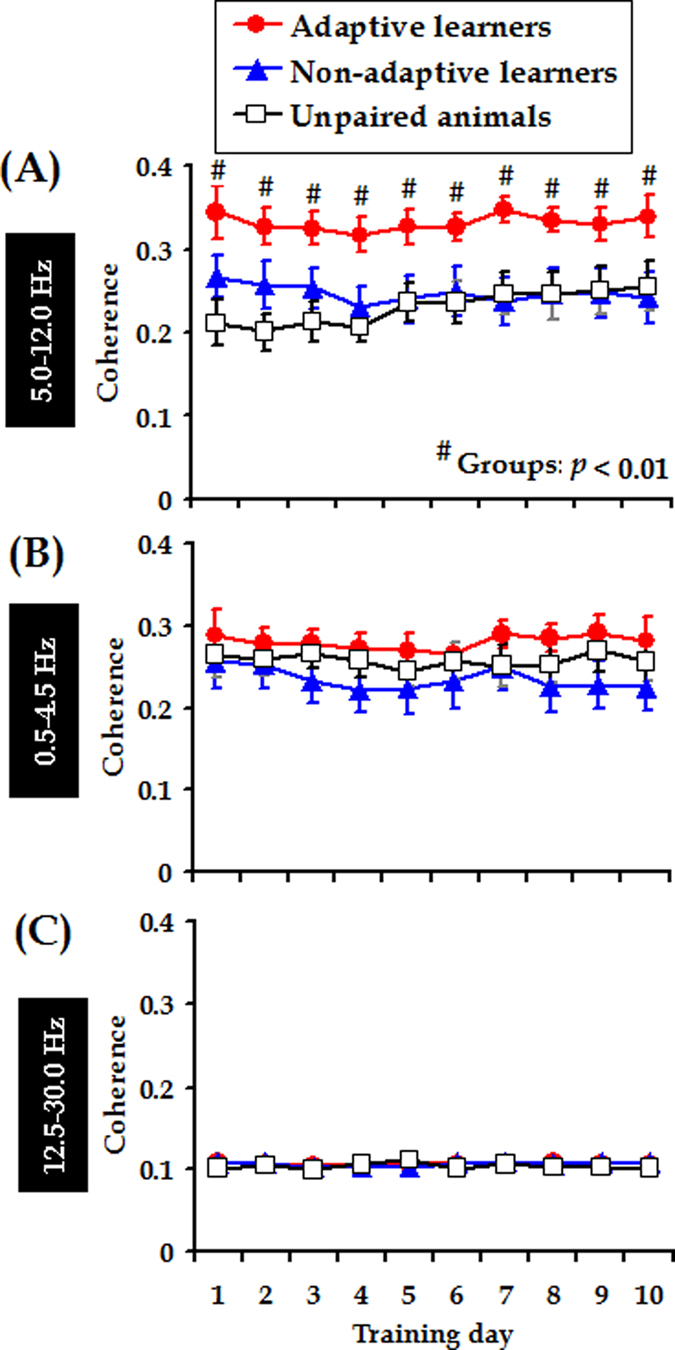
mPFC-cerebellum theta-band synchrony remains stable across training, but is stronger during the performance of adaptive CRs. (**A**) Stronger mPFC-cerebellum theta-band (5.0–12.0 Hz) coherence was consistently observed in the adaptive learners (*n* = 11, red circles) compared with the non-adaptive learners (*n* = 12, blue triangles) and unpaired animals (*n* = 11, open squares). Nevertheless, mPFC- cerebellum coherence was comparable among three groups of animals in the delta-band (**B**, 0.5–4.5 Hz) and beta-band (**C**, 12.5–30.0 Hz). Data are given as the mean ± SEM. Symbols are the same for (**A**–**C**).

It was found that theta coherence between the mPFC and the cerebellum remained relatively stable across 10 training days. This notion was demonstrated by statistical results that showing no obvious effects of training days on theta coherence in all experimental groups (*F*_[9, 279]_ = 1.053, *p* = 0.398, Fig. 5A). Nevertheless, during the acquisition, stronger mPFC-cerebellum theta-band synchronization was revealed in the adaptive learners than in the non-adaptive learners and the unpaired animals. Two-way ANOVA with repeated measures on coherence in the theta range revealed a significant effect of group (*F*_[2, 31]_ = 6.400, *p* = 0.005, Day of training X Group interaction: *F*_[18, 279]_ = 0.912, *p* = 0.565; *post hoc* (LSD): adaptive *vs*. non-adaptive, *p* = 0.008; adaptive *vs*. unpaired, *p* = 0.002; non-adaptive *vs*. unpaired, *p* = 0.576, Fig. 5A). In contrast, no statistically significant differences in coherence at any other frequency band (Delta band: 0.5–4.5 Hz and Beta band: 12.5–30.0 Hz) were found between the mPFC and the cerebellum (Fig. 5B,C, both *p*_s_ > 0.05). These results suggested that stronger synchronization between the mPFC and the cerebellum occurred specifically to occur in the adaptive learners and in the theta-frequency (5.0–12.0 Hz) range.

To further ascertain whether theta-band synchronization occurred specifically between the mPFC and the cerebellum, we evaluated mPFC-HIP theta coherence in another group of animals acquiring adaptive CRs (Fig. S4A,B). It was revealed that theta-band coherence between the mPFC and hippocampus was clearly lower than between the mPFC and cerebellum (*F*_[1, 21]_ = 8.325, *p* = 0.009, interaction between Day of Training and Group, *F*_[9, 189]_ = 0.192, *p* = 0.995, Fig. S4C). However, no such difference was revealed in either the delta-band (*F*_[1, 21]_ = 1.664, *p* = 0.211, Fig. S4D) or the beta-band (*F*_[1, 21]_ = 0.019, *p* < 0.891, Fig. S4E). Therefore, our results indicate that theta-band synchronization occur specifically between the mPFC and the cerebellum.

### Stronger mPFC-cerebellum synchrony was due to temporal alignment of rhythmic theta activities

**Figure 6:**
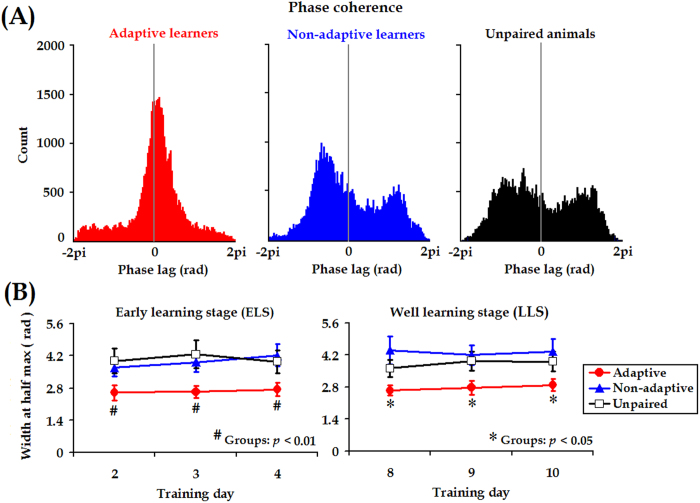
Theta-band phase coherence in the early- and late-learning stages. (**A**) Representative histogram of theta-band phase differences. Instantaneous theta phase of two LFP signals were subtracted from each other, and differences in theta phase between the mPFC-cerebellum were plotted as a histogram in an adaptive learner, a non-adaptive learner, and an unpaired animal. (**B**) Width of theta-band phase difference histogram at half the peak height averaged across animals for mPFC-cerebellum in the adaptive learners (*n* = 11, red circles), non-adaptive learners (*n* = 12, blue triangles) and unpaired animals (*n* = 11, open squares) in the early learning stage (ELS, days 2–4) and the late learning stage (LLS, days 8–10). Data are given as the mean ± SEM.

**Figure 7:**
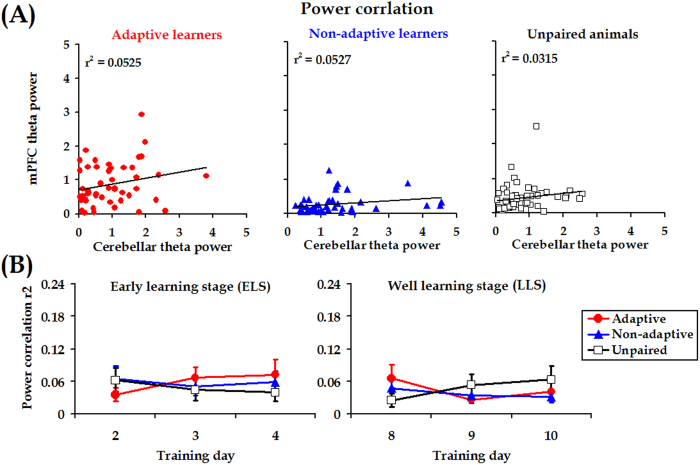
Theta-band power correlation in the early- and late-learning stages. (**A**) Representative examples of theta power correlation scatter plots for mPFC-cerebellum in an adaptive learner, a non-adaptive learner and an unpaired learner from a conditioning training session. (**B**) Averages of linear correlation coefficients of theta-band power across animals in the adaptive learners (*n* = 11, red circles), non-adaptive learners (*n* = 12, blue triangles) and unpaired animals (*n* = 11, open squares) in the early learning stage (ELS, days 2–4) and late learning stage (LLS, days 8–10). Data are given as the mean ± SEM.

Higher theta-band (5.0–12.0 Hz) coherence between two LFP signals indicated stronger synchronization but did not disambiguate whether the stronger synchronization was due to correlated fluctuation in power (related to oscillation amplitude) or due to the constant phase relationship (related to oscillation timing). To further ascertain the mechanisms underlying the stronger theta-band coherence between the mPFC and cerebellum in the adaptive learners, we separately measured phase coherence and power correlation in the theta-frequency range. As previously described by Adhikari *et al.*^45^, theta phase coherence could be estimated by calculating a histogram of the differences in instantaneous phase between two LFP signals and measuring the width at half of the histogram peak. It was found that, in the theta range, phase coherence in the adaptive learners was significantly higher than in the non-adaptive learners and the unpaired animals, as shown by the narrower peak of the phase difference histogram in the early learning stage (ELS; [days 2–4]: *F*_[2, 31]_ = 7.081, *p* = 0.003, Day of training X Group interaction: *F*_[4, 62]_ = 0.533, *p* = 0.712, Fig. 6A,B left panel) or in the late learning stage (LLS; [days 8–10]: *F*_[2, 31]_ = 4.259, *p* = 0.023, Group X Day of training interaction: *F*_[4, 62]_ = 1.324, *p* = 0.271, Fig. 6B right panel). In contrast, theta power correlation between the mPFC and cerebellum was statistically comparable among the three groups (ELS: *F*_[2, 31]_ = 2.576, *p* = 0.092, Fig. 7A,B left panel, LLS: *F*_[2, 30]_ = 0.100, *p* = 0.905, Fig. 7B right panel). In addition, the mean coefficient *r*^2^ value (~0.045) suggested that the relative theta power of the signal in the mPFC was unlikely to co-vary with corresponding LFP from the cerebellum. Collectively, our current measurements suggested that stronger theta-band synchronization in the adaptive learners most likely arose from temporal alignment of theta-rhythmic neural activities in the mPFC and the cerebellum.

### Time-limited association of mPFC-cerebellum theta synchrony with adaptive CR performance

**Figure 8:**
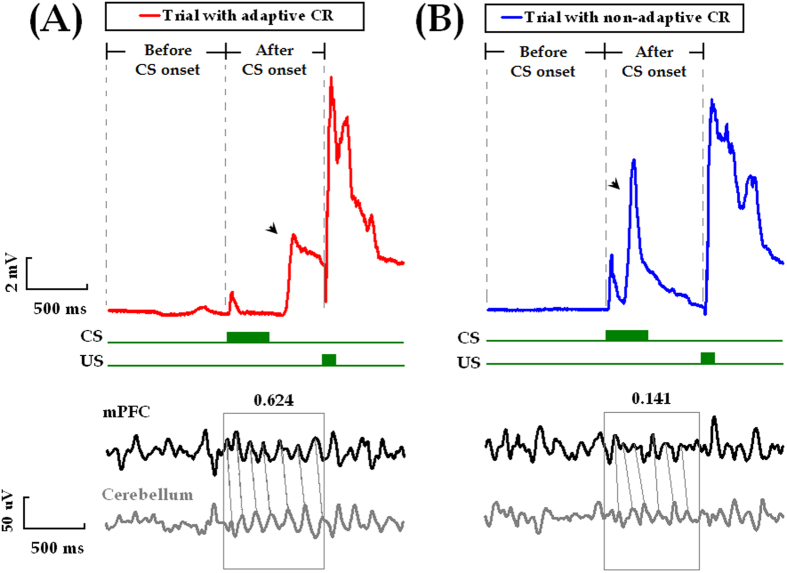
mPFC-cerebellum theta-band coherence was higher when adaptive CR occurred. Representative raw LFP signals simultaneously recorded from the mPFC (black traces) and the cerebellum (gray traces) during performance of an adaptive CR (**A**) and a non-adaptive CR (**B**), respectively. Horizontal scale bar indicates 0.5 sec. Vertical scale bar indicates 2 mV for the eyelid movements and 50 μV for the LFP signals.

**Figure 9:**
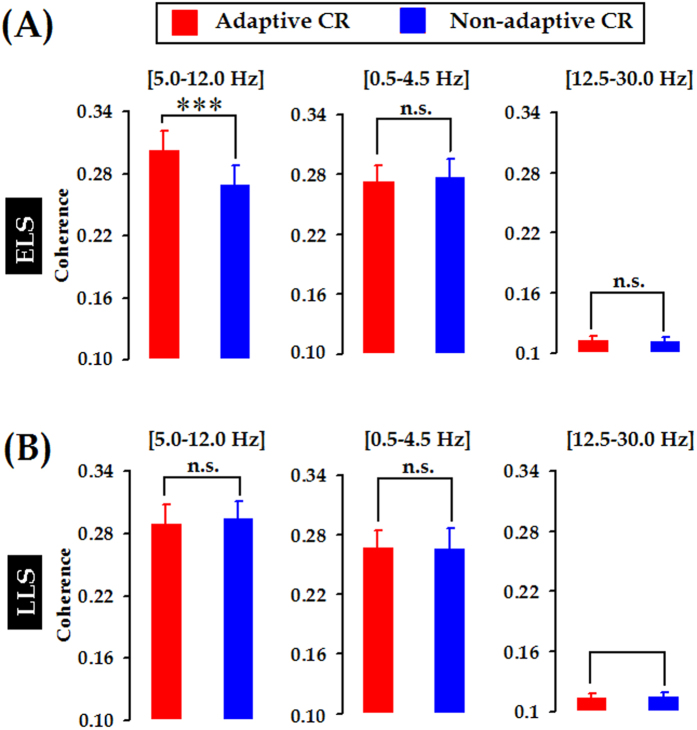
Time-limited association of mPFC-cerebellum theta coherence with adaptive CR performance. (**A**) In the early learning stage (ELS, days 2–4), mPFC-cerebellum theta-band (5.0–12.0 Hz) coherence was specifically higher in the trials where adaptive trace CRs occurred. By contrast, no such differences were found in the delta- and beta-frequency bands. (**B**)In the late learning stage (LLS, days 8–10), no differences were observed in either frequency band. Data are given as the mean ± SEM. Symbols are the same for (**A**,**B**).

Thus far, the function of mPFC-cerebellum theta-band synchronization remained unclear ? To address this question, we further quantified the correlation between the mPFC-cerebellum theta-band coherence and the CR performance trial-by-trial. Intriguingly, it was revealed that, during the ELS (days 2–4), theta-band coherence was specifically higher in the trial where the adaptive CR occurred (*t*_(22)_ = 4.073, *p* < 0.001 for paired *t* test, Figs 8 and 9A left panel, and Fig. S5). In contrast, no obvious association of coherence in any other frequency band with adaptive CR performance was revealed (Delta band: 0.5–4.5 Hz, *t*_(22)_ = −0.578; Beta band: 12.5–30.0 Hz, *t*_(22)_ = 0.425; both *p*_*s*_ > 0.05 for paired *t* tests, Fig. 9A middle and right panels). Given that the adaptive learners manifested similar UR and SR timing to the non-adaptive learners, our results supported the idea that the adaptive performance of trace CRs might be specifically associated with coherent mPFC-cerebellum theta oscillations.

Interestingly, no obvious association of theta-band coherence with adaptive CR performance was observed in the LLS (days 8–10, *t*_(22)_ = −0.487, *p* = 0.631 for paired- sample *t* tests, Fig. 9B left panel). Similarly, in the LLS, the association of coherence with adaptive CR performance did not reach the level of significance in either the delta- or the beta-band (Delta band, 0.5–4.5 Hz: *t*_(22)_ = 0.087; Beta band, 12.5–30.0 Hz: *t*_(22)_ = −0.403; both *p*_*s*_ > 0.05 for paired-sample *t* tests, Fig. 9B middle and right panels). These results suggested that the association of adaptive CR performance with mPFC-cerebellum theta synchrony might be time limited.

**Figure 10:**
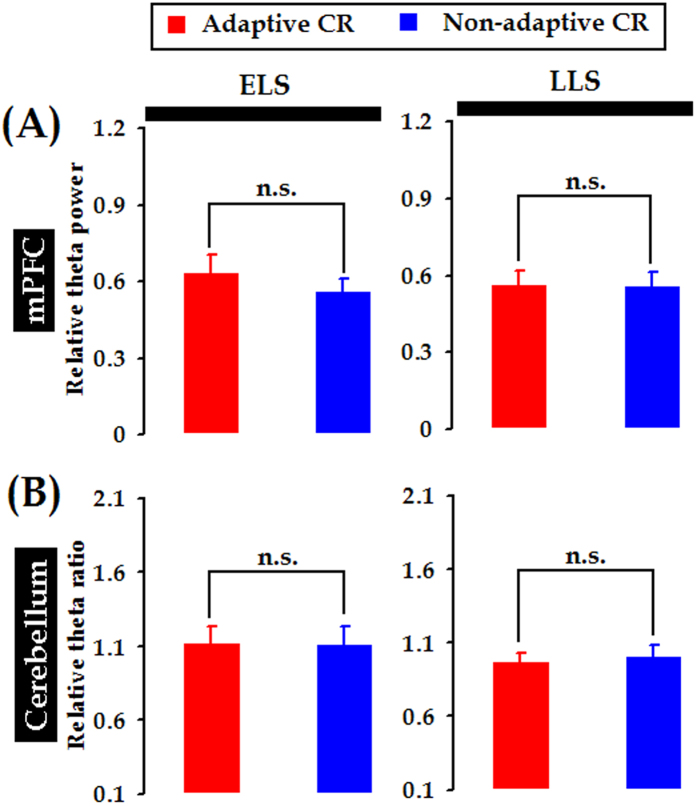
Relative theta power in neither the mPFC nor the cerebellum was associated with adaptive CR performance. (**A**) In both the ELS (days 2–4) and the LLS (days 8–10), there were no obvious differences in prefrontal relative theta power when adaptive and non-adaptive CRs occurred. (**B**) Likewise, in both the ELS and the LLS, there were no apparent differences in cerebellar theta power when the adaptive and non-adaptive CRs were observed. Data are given as the mean ± SEM. Symbols are the same for (**A**,**B**).

Given that the theta power in the mPFC of adaptive learners was stronger (Fig. S6), we also examined whether the relative theta power in the mPFC was associated with CR performance. The statistical analysis revealed no apparent association of theta power in the mPFC with CR performance in either the ELS or the LLS (ELS: *t*_(22)_ = 1.901; LLS: *t*_(22)_ = 0.313; both *p*_*s*_ > 0.05 for paired-sample *t* tests, Fig. 10A). Likewise, no association of relative theta power in the cerebellum with CR performance was found (ELS: *t*_(22)_ = 0.166; LLS: *t*_(22)_ = −0.710; both *p*_*s*_ > 0.05 for paired-sample *t* tests, Fig. 10B). Taken together, our results suggest that the adaptive CR performance was associated with mPFC-cerebellum theta synchrony instead of relative theta power in both areas.

### Disruption of mPFC-Cerebellum theta synchronization impaired adaptive CR performance

If the coherent phase relationship between the mPFC and the cerebellum provides a possible mechanism by which the two areas cooperate during TEBC, the performance of CRs might be expected to co-vary with theta-band coherence. Hypothalamic orexinergic fibers have been reported to comprise the third class of cerebellar afferent inputs, which participate in the modulation of neuronal excitability rather than in the transmission of sensory and motor information in the cerebellum^46^,^47^. Thus, it was reasonable to expect that injections of SB-334867, a selective orexin receptor-1 antagonist, would influence rhythmic LFP activity in the cerebellum, subsequently disrupting theta-band coherence between the mPFC and the cerebellum^35^. In this way, we tested whether the disrupted mPFC-cerebellum theta coherence would be accompanied by the impairment of CR performance.

**Figure 11:**
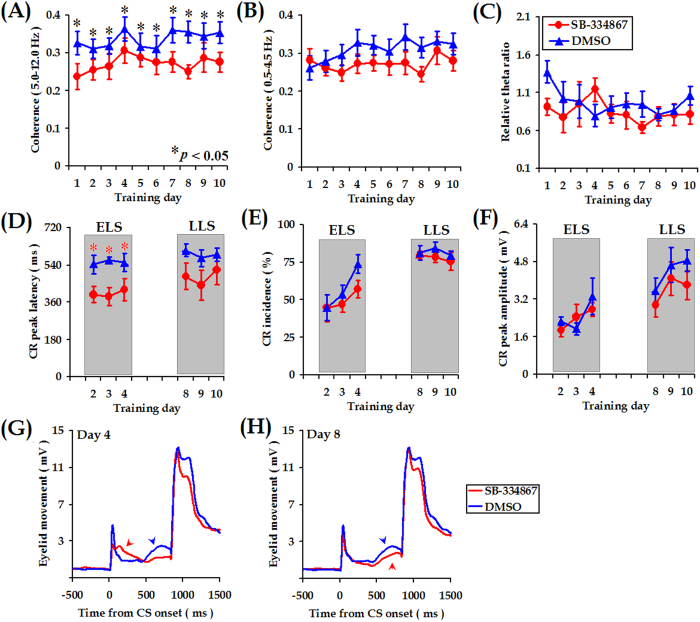
Blockade of orexinergic receptor-1 in the cerebellum disrupted both mPFC- cerebellum theta-band coherence and adaptive CR performance. (**A,B**): Injections of SB-334867 into the intermediate cerebellum decreased mPFC-cerebellum coherence in theta-band (5.0–12.0 Hz) instead of in the delta-band (0.5–4.5 Hz). (**C**) Relative theta power in the cerebellum was not significantly attenuated by the SB-334867 injections. (**D**) Compared with DMSO (*n* = 7, red circles), the SB-334867 injections (*n* = 7, blue triangles) decreased the latency to CR peak in the early learning stage (ELS, days 2–4). (**E**,**F**) The SB-334867 injections seemed not to reduce the incidence and magnitude of trace CRs. Data are shown as mean ± SEM. Symbols are the same for (**A**–**F**). The averaged effects of SB-334867 (black traces) and DMSO (gray traces) injections on the CR performance on days 4 and 8 are illustrated, respectively.

In this set of experiments, the effectiveness of injections was histologically and functionally examined (Figs S7 and S8). It was found that, in comparison with DMSO injections, injections of SB-334867 into the left intermediate cerebellum decreased mPFC-cerebellum coherence in the theta-frequency band (*F*_(1, 12)_ = 4.589, *p* = 0.049, Fig. 11A; Group X Day of training interaction: *F*_(9, 108)_ = 0.576, *p* = 0.814) but not in the delta-frequency band (*F*_(1, 12)_ = 1.391, *p* = 0.261, Fig. 11B). Moreover, decreased theta-band coherence was not likely to result from the disruption of relative theta power in the cerebellum, because theta power was comparable between the SB-334867 and DMSO injection groups across 10 training days (*F*_(1, 12)_ = 0.595, *p* = 0.456, Fig. 11C). These results suggest that functional disruption of the orexin receptor-1 in the cerebellum decreased mPFC-cerebellum theta coherence instead of relative power in the same frequency band.

As expected, accompanying with the decreased mPFC-cerebellum theta coherence, the adaptive performance of trace CRs was impaired by the SB-334867injections. This notion was supported by our statistical results that, compared with the animals receiving the injections of DMSO, the animals receiving SB-334867 injections showed earlier CR peak during the ELS (*F*_(1, 12)_ = 13.158, *p* = 0.003, Groups X Day of training interaction, *p* > 0.05, Fig. 11D,G). In contrast to the impaired timing of CRs, however, the SB-334867 injections seemed not to significantly influence the CR acquisition (Incidence: *F*_(1, 12)_ = 0.916, *p* = 0.358, Magnitude: *F*_(1, 12)_ = 0.084, *p* = 0.777, Fig. 11E,F). Additionally, the SB-334867 injections failed to shift forward the peak latency of trace CRs during the LLS (*F*_(1, 12)_ = 2.328, *p* = 0.153, Fig. 11D,H). Taken together, these results suggest a time-limited correlation of decreased mPFC-cerebellum theta coherence with impaired CR performance.

## Discussion

Accumulating evidence has suggested that TEBC requires cooperation of the mPFC with the cerebellum. However, the neural mechanisms by which these two areas cooperate are not well-known. We show evidence that, in the theta-frequency (5.0–12.0 Hz) range, LFP signals recorded from the mPFC are phase-locked to LFP signals recorded in the cerebellum during TEBC. The coherent mPFC-cerebellum theta-band oscillation was associated with the adaptive performance of trace CR especially during the ELS. Finally, a decrease in mPFC-cerebellum theta-band coherence was accompanied by impaired adaptive CR performance. These findings thus support the idea that, in a long-range neural network, the mPFC and the cerebellum may work together to contribute to adaptive associative learning behavior by means of theta-band synchronization.

Theta rhythm is one of the typical neural activities that support neural communication and synaptic plasticity in the brain^48^. In this study, we found that CS-evoked theta-frequency (5.0–12.0 Hz) oscillations appeared in both the mPFC and the cerebellum, as illustrated in Fig. 3. Importantly, to the best of our knowledge, we have presented the first evidence that theta oscillation in the cerebellum is consistently phase-locked to theta oscillation in the mPFC (Figs 3B and 4D) during TEBC. This phase-locking effect is unlikely to have resulted from volume conduction because obvious phase lag was observed between the two LFP signals, as shown in Figs 3B and 6. Moreover, cerebellar theta power decreased across conditioning training (Fig. S6B), whereas power of mPFC theta activity remained relatively stable (Fig. S6A). As a result, the mPFC-cerebellum theta synchronization was unlikely to be account for co-varying theta power between the two areas (Fig. 7). Instead, it might arise from temporal alignment of theta-band phase (Fig. 6). Considering theta-frequency resonance in cerebellar granule cells, together with location of the recording sites close to the cerebellar cortex, it is reasonable to propose that, during TEBC, the cerebellum may respond to theta-frequency rhythmic inputs from the mPFC, and align its oscillation activity with the mPFC. Given that cooperation between the mPFC and cerebellum has been implicated^3^,^4^, our results suggested that the mPFC-cerebellum cooperation, to some extent, is reflected by theta-band synchronization between two areas.

It also should be noted that theta oscillations recorded from the mPFC and the cerebellum showed phase reversal after the CS onset. One possible explanation for this result is the tone CS evoked significantly increased discharge in the mPFC neurons, which led to transfer of positive electrical charge (e.g., sodium) into the intracellular environment. This transfer thus induced negative fluctuation of LFP relative to the reference sites. In the meanwhile, due to long-term depression (LTD) in the parallel fiber-Purkinje cell, the CS evoked decreased (even paused) discharge of Purkinje cells. Given its high-frequency firing, decrease of Purkinje cell activity might lead to positive LFP fluctuation. However, this explanation should be tested in future experiments by means of simultaneously multiple units recording in both two areas during TEBC.

In light of previous observations that theta synchronization between the mPFC and the hippocampus^28^,^29^,^48^, the V4 area of visual cortex^31^, and the posterior association cortex^30^ are associated with various learning and memory processes, we have proposed that formation of a transient theta-synchronized assembly of the mPFC and the cerebellum might be correlated with memory performance in associative learning behavior. This idea was supported by the findings that the adaptive CR occurred specifically in the trial when mPFC-cerebellum theta synchrony was stronger (Figs 8 and 9A). However, the mPFC-cerebellum theta-band synchrony failed to become correlated with CR acquisition because our results showed that the mPFC-cerebellum theta coherence remained stable across conditioning training (Fig. 5), whereas the incidence and magnitude of CRs increased (Fig. 2). This result was unexpected because both the mPFC^12^,^13^,^14^,^50^,^51^ and the cerebellum^52^,^53^ have long been demonstrated to be involved in the acquisition of trace CRs. Together with previously observed constant hippocampal-cerebellar theta-band synchrony during TEBC^34^, one possible explanation for our results is that theta synchronization might serve as a fundamental and stable mediator of neural communication between the mPFC and the cerebellum.

Central to our study is the investigation how strongly mPFC-cerebellum theta synchrony contributes to the adaptive CR performance. As previously reported, the mPFC could play a definite role in the expression of newly acquired CRs^54^. Furthermore, in a trace conditioning task, it has been revealed that theta oscillation can synchronize neuronal activities at the distributed mPFC sites during trace conditioning task, leading to more effective activation in the downstream structure^32^. At the signal input stage of the cerebellum, granular cells have been revealed to generate their maximum resonance response at the theta-frequency band *in vivo* following sensory stimulation and *in vitro* following mossy fiber stimulation^36^,^37^. It has also been documented that processing in the granular layer is critical for controlling the timing of associative learning behaviors^55^. These points, together with theta-band oscillation’s relatively slow time scale allowing long synaptic delay^48^,^49^, support the current hypothesis that a higher level of theta synchronization might promote mPFC-cerebellum communication and granular layer processing, thus contributing to the adaptive CR expression during TEBC. One effective way to test this hypothesis is to examine whether neuronal firing in the cerebellum is phase- locked to theta activity in the mPFC, and whether spike-field theta coherence contributes to the adaptive CR performance.

Interestingly, the association of mPFC-cerebellum theta-band synchrony with adaptive CR performance seems to be time-limited, as suggested by the findings that mPFC-cerebellum theta coherence was associated with the adaptive performance of trace CRs only in the early learning stage and not after an asymptotic CR level had been achieved (Fig. 9). Accordingly, a decrease in mPFC-cerebellum theta coherence was accompanied by impaired CR timing in the ELS rather than in the LLS (Fig. 11D,G). These findings are at odds with the involvement of the mPFC and the cerebellum in the acquisition, retention, and consolidation of trace CRs^12^,^13^,^14^,^40^,^52^,^53^,^56^. Notably, in this study, the majority of electrodes were located in the ACC area of mPFC (Fig. S2). It has been reported that tone CS-elicited increase in the ACC neuronal activity persisted throughout the initial few days of TEBC and gradually declined during subsequent training^20^,^24^. Together with the involvement of ACC in modulating the CR timing^57^, we proposed that, as a fundamental and stable mediator of communication among the distributed brain regions, stronger theta-band synchronization has the advantage of transmitting learning stage-specific neuronal responses from the ACC to the cerebellum, leading to a time-limited association of mPFC-cerebellum theta coherence with adaptive CR performance. It also should be noted that, about 20% recording electrode tip were located in the PL area of mPFC. Association theta coherence with CR performance was not separately analyzed between the ACC and PL, because few number of PL electrode tips in the present study (adaptive leaners, n = 1, non-adaptive learners, n = 3, respectively). However, Hattori *et al.* provided evidence that single neuron recordings in the PL exhibited much greater activity related to the CR after the consolidation of TEBC, whereas the ACC neurons increased early in learning and decreased as the learning progressed^24^. Consequently, further experiments are needed to test whether the PL area of mPFC would show greater theta coherence later in learning, especially following memory consolidation.

Additionally, we did not observe obvious theta synchronization between the mPFC and hippocampus during TEBC (Fig. S4). This result was unexpected because theta synchronization between the cerebellum and hippocampus has been reported^33^,^34^. Logically, it is reasonable to propose there should be synchronization between the mPFC and hippocampus. Given that frequency range of theta oscillation in the mPFC is differential from that in the hippocampus during the trace conditioning task^32^,^33^,^34^, one possible explanation for the discrepancy was the different theta-frequency band we analyzed (5–12 Hz) compared with the previous studies (3–7 Hz for Hoffmann *et al.* and 6 Hz for Wikgren *et al.*)^33^,^34^. Additional experiments should be done to test whether there is synchronization between the mPFC and hippocampus in specific frequency range.

The function of the cerebellum has been documented to be under the influence of various neuropeptides. The orexins (including A and B), among these neuropeptides, are exclusively produced by a specific group of neurons located in the lateral hypothalamus, and widely released in the central nervous system^58^. It has also been documented that the cerebellum receives orexinergic innervations from the lateral hypothalamus^59^,^60^. Moreover, application of the exogenous orexins to the rat cerebellar slices led to increased neuronal excitability^46^,^60^. Extending from the anatomical and electrophysiological data, we have shown that functional inactivations of the orexin receptor-1 in the cerebellum with SB-334867 disrupt local theta oscillations^35^. Consequently, in this study, we further attempted to disrupt mPFC-cerebellum theta coherence by injection of SB-334867 into the cerebellum. The data confirmed the validity of theta coherence disruption by the cerebellar SB-334867 injection, highlighting the influence of endogenous orexins on mPFC-cerebellum theta-band synchronization. Additionally, our experiments with the SB-334867 injection indicate the presence of mechanism that mediates interaction between the mPFC and the cerebellum.

In relation to one technical issue, it might be argued that enhancement in theta-band synchronization was induced by eyelid movement (i.e., generation of CR). This possibility was unlikely to play a major role in the observed theta-band synchronization, because the adaptive learners exhibited CRs with incidence and amplitude similar to the non-adaptive learners across conditioning training (Fig. 2A,B). In addition, the theta coherence of the adaptive learners was higher than in the non-adaptive learners even on training day 1 (Fig. 5). If the synchronized LFP signals were heavily contaminated with signals that originated from the eyelid movements, these results would not be possible, because there were no apparent CRs in either the adaptive or non-adaptive group of animals on training day 1 (Fig. 2A).

In summary, our current findings indicate that mPFC-cerebellum theta synchronization is associated with the adaptive performance of trace CRs, especially during the early stage of associative learning. Given that learning to regulate the accurate occurrence timing of motor responses is one of the most important properties of cerebellum, our results shed new lights on how the mPFC cooperates with the cerebellum during adaptively performing associative learning behavior.

## Additional Information

**How to cite this article**: Chen, H. *et al.* Theta synchronization between medial prefrontal cortex and cerebellum is associated with adaptive performance of associative learning behavior. *Sci. Rep.* **6**, 20960; doi: 10.1038/srep20960 (2016).

## Supplementary Material

Supplementary Information
