# Supplementary material for: Theta synchronization between medial prefrontal cortex and cerebellum is associated with adaptive performance of associative learning behavior
Source: Sci Rep. 2016 Feb 16;6:20960. doi: 10.1038/srep20960 (PMC4754690; doi:10.1038/srep20960)
Supplement: Supplementary Information [file srep20960-s1.pdf]

Supporting Online Material for

Theta synchronization between medial prefrontal cortex and cerebellum is associated with adaptive performance of associative motor behavior

Hao Chen, Yi-jie Wang, Li Yang, Jian-feng Sui, Zhi-an Hu, Bo Hu

To whom correspondence should be addressed:

E-mail: bohu@tmmu.edu.cn (B. Hu)

This file includes 1 table and 8 figures.

Suppl. Table 1 Summary of non-significant differences in topography of UR and SR between adaptive and non-adaptive learners, related to Figure 2.

Suppl. Figure 1 Independence of theta coherence from theta power.

Suppl. Figure 2 Electrode placements, related to Figure 3.

Suppl. Figure 3 Theta phase differences during the 850-ms period before CS onset and 850-ms period after CS onset, related to Figure 5.

Suppl. Figure 4 Phase coherence between mPFC and hippocampus (HIP) in adaptive learners, related to Figure 5.

Suppl. Figure 5 Independent analysis of association between mPFC-cerebellum theta coherence and adaptive CR performance in two subgroups of animals, related to Figure 9.

Suppl. Figure 6 Relative theta power in mPFC and cerebellum after 850-ms period after CS onset across ten training days, related to Figure 10.

Suppl. Figure 7 Histological examination of cannula tips in the cerebellum, related to Figure 11.

Suppl. Figure 8 Effects of injections of muscimol into the cerebellum on the expression of acquired trace CRs on day 11, related to Figure 11.

Table S1 Summary of non-significant differences in the topography of UR and SR between adaptive and non-adaptive learners.

**Topographical parameters**	**Comparison of means across ten conditioning days**
UR peak amplitude (mV)	Two-way ANOVA with repeated measures: $F_{(1, 21)} = 0.144$, $p = 0.708$
UR peak latency (ms)	Two-way ANOVA with repeated measures: $F_{(1, 21)} = 2.\ 160$, $p = 0.156$
SR peak amplitude (mV)	Two-way ANOVA with repeated measures: $F_{(1, 21)} = 0.392$, $p = 0.538$
SR peak latency (ms)	Two-way ANOVA with repeated measures: $F_{(1, 21)} = 0.016$, $p = 0.899$
SR onset latency (ms)	Two-way ANOVA with repeated measures: $F_{(1, 21)} = 0.019$, $p = 0.892$

Figure_S1

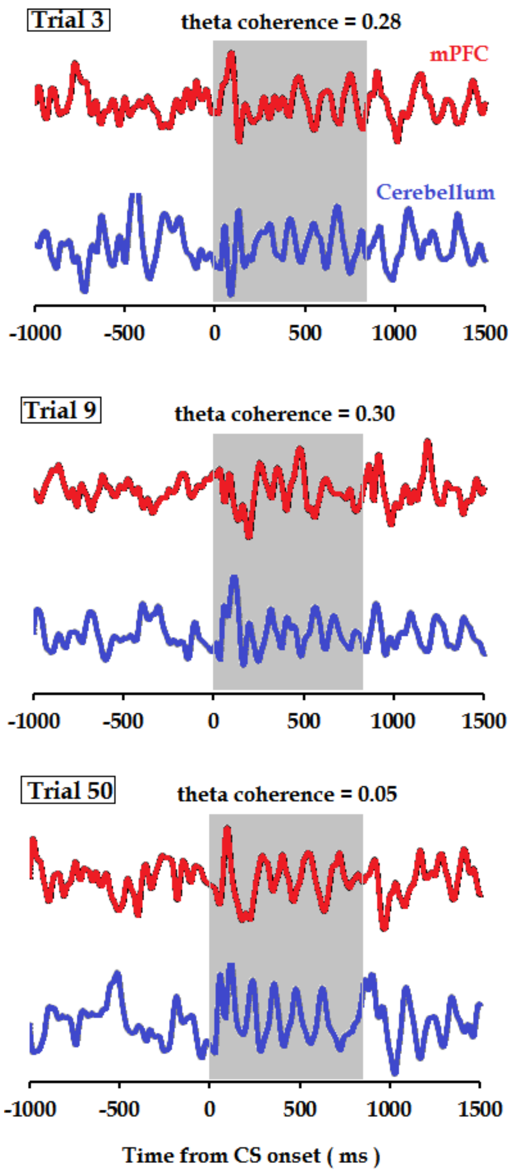

Figure S1 | Independence of theta coherence from theta power. It was revealed that low level of theta coherence occurred between signals recorded from the mPFC (red trace) and the cerebellum (blue traces), where both signals showed strong theta oscillations. All the signals were obtained from a representative guinea pig on training day 3.

Figure_S2

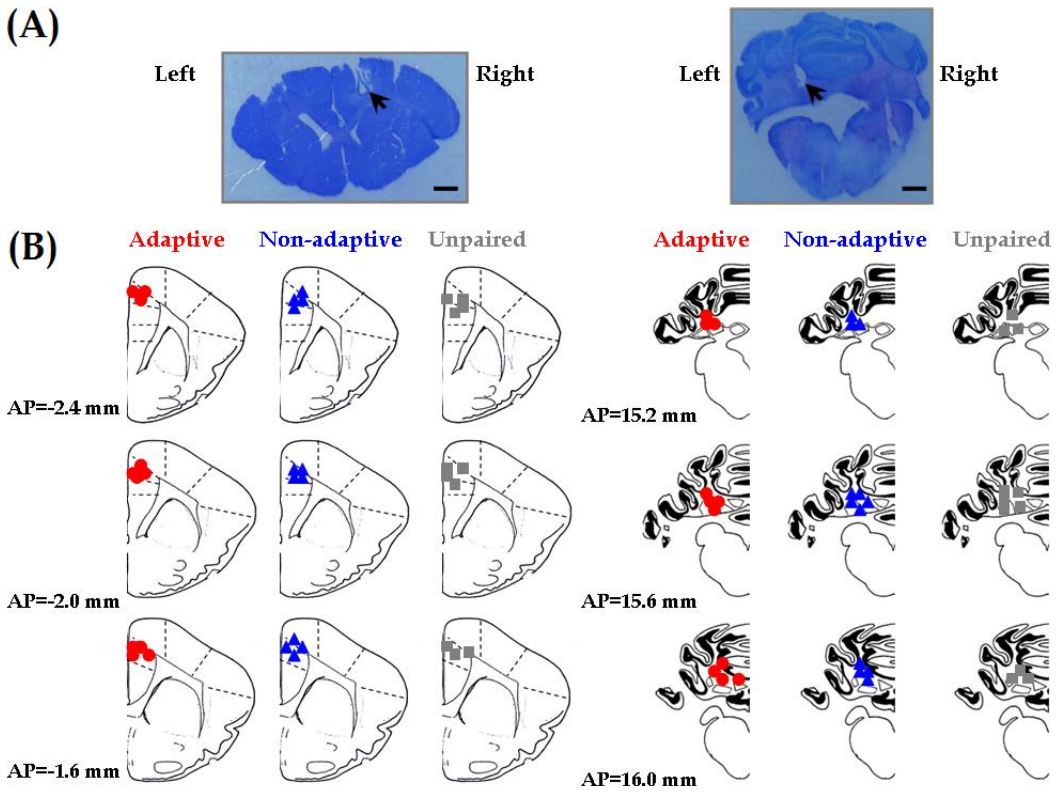

Figure S2| Electrode placements. (A) Representative toluidine blue-stained coronal slices showing lesions (indicated by the black arrows) marking electrode tip locations in the mPFC and the cerebellum. **(B)** Line drawings of coronal sections showing the electrode placements in adaptive learners (n = 11, red circles), non-adaptive learners (n = 12, blue triangles), and unpaired animals (n = 10, gray squares) in the mPFC and the cerebellum, respectively. ***Right panel***: The black regions represent the granular cell layer (GCL) of cerebellar cortex. Numbers represent the distance (mm) between the sections and Bregma.

Figure_S3

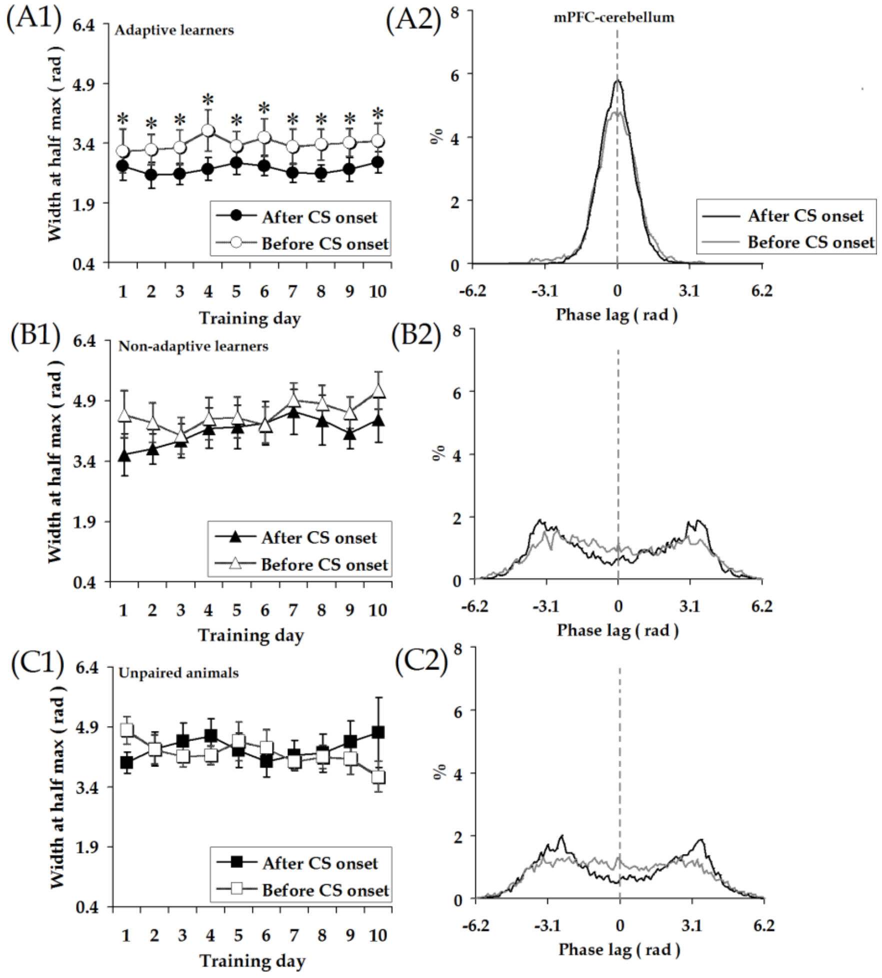

Figure S3 | Theta phase differences during 850-ms period before CS onset (baseline) and 850-ms period after CS onset. (A1-C1) Width of theta phase difference histogram at half the peak height across adaptive learners (*n* = 11, circles), non-adaptive learners (*n* = 12, triangles), and unpaired animals (*n* = 10, squares). The statistical analysis revealed that, in the adaptive learners, the width at half maximum height after the CS onset was significantly narrower than before the CS onset ($F_{(1,10)} = 88.270$, $p < 0.001$, Day of Training X Group interaction: $F_{(9, 189)} = 0.542$, $p = 0.842$; two-way ANOVA with repeated measures, indicated by asterisks). Such decreases were not revealed in either the non-adaptive learners or the unpaired animals. **(A2-C2**) Representative histogram of theta phase difference. Instantaneous theta phase of two LFP signals from the mPFC and the cerebellum were subtracted from each other and the differences in theta-band phase were plotted as a histogram. Narrower peaks in the histogram indicate a more consistent phase relationship.

Figure_S4

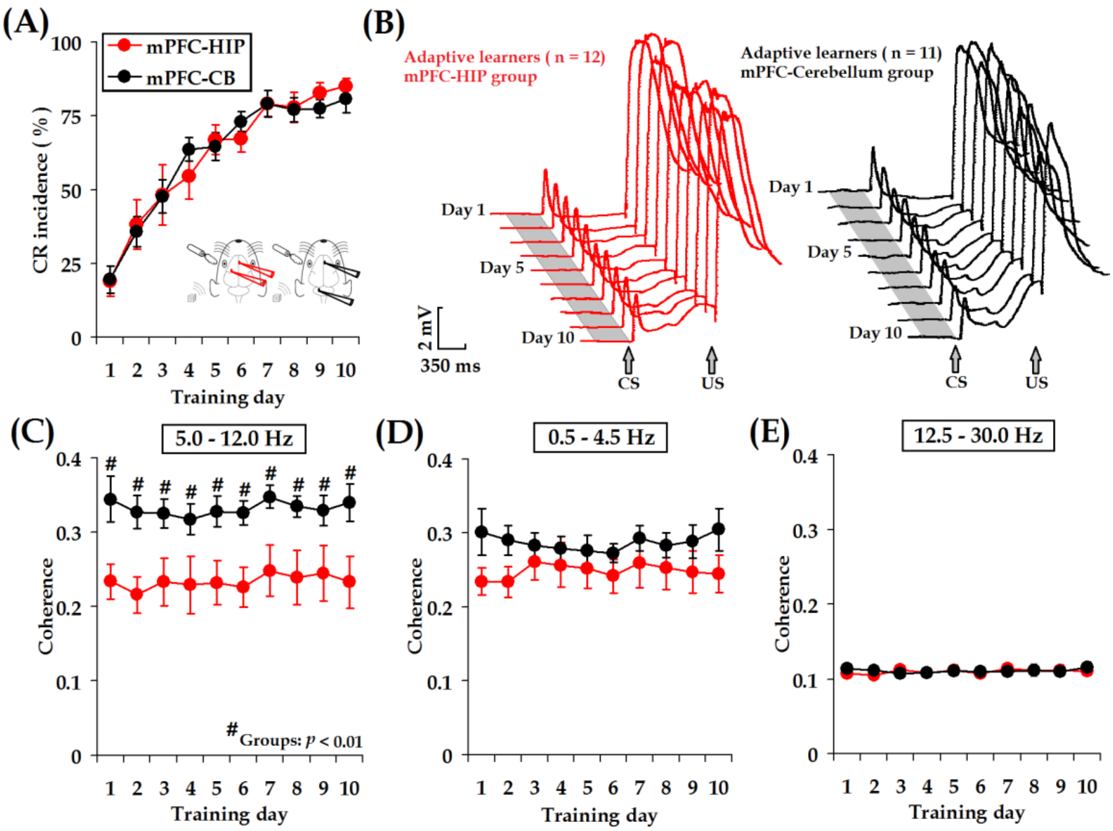

Figure S4 | The mPFC-hippocampus (HIP) phase coherence across ten training days. (A) CR acquisition in mPFC-HIP (*n*= 12, red) and mPFC-cerebellum (*n*= 11, black) animals. It was shown that the two groups of animals emitted the same level of trace CRs across ten training days. **(B)** Both groups of animals showed obvious adaptive CRs after learning. **(C) A** higher level of theta-band (5.0-12.0 Hz) coherence was observed in the mPFC-cerebellum animals than in the mPFC-HIP animals. However, no differences in delta- **(D**, 0.5-4.5Hz) and beta-band **(E**, 12.5-30.0 Hz) between the mPFC-cerebellum and mPFC-HIP animals were observed. Symbols are the same for **(A)** and **(C)-(E)**.

Figure_S5

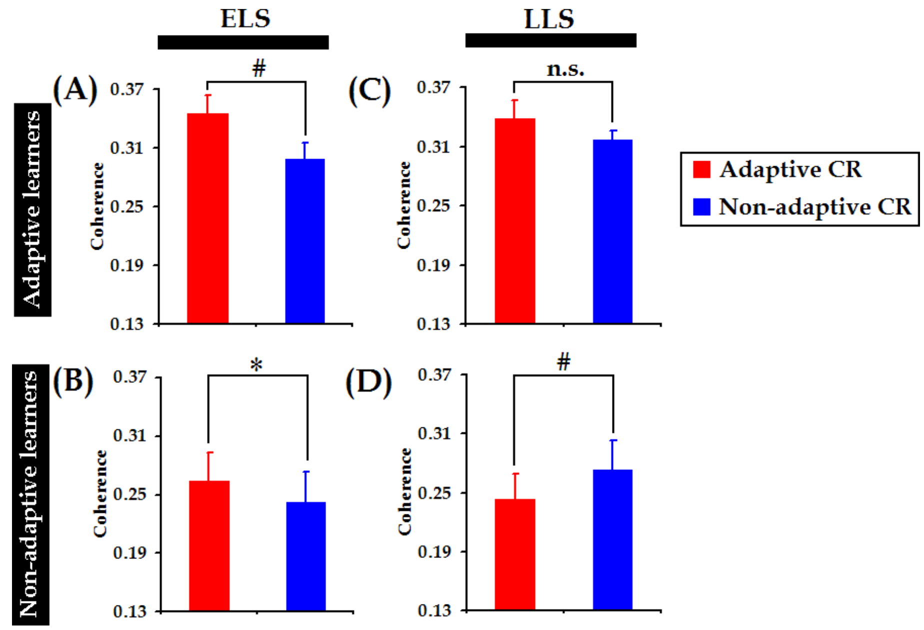

Figure S5 | Independent analysis of association between mPFC-cerebellum theta coherence and adaptive CR performance in the two subgroups of animals. During the ELS (days 2-4), the adaptive CR occurred preferentially in the trial where theta coherence was higher in either the adaptive (**A**, n = 11, $t_{(10)}$ = 4.248, p = 0.002, Paired-sample t test) or the non- adaptive learners (**B**, n = 12, $t_{(11)}$ = 2.208, p = 0.049). During the LLS (days 8-10), there was no significant correlation between theta coherence and CR performance in the adaptive learners (**C**, $t_{(10)}$ = 1.073, p= 0.309, Paired-sample t test). In contrast, adaptive CR occurred specifically in the trial where theta coherence was lower in the non-adaptive learners (**D**, $t_{(11)}$ = -3.692, p = 0.004, Paired-sample t test).

Figure_S6

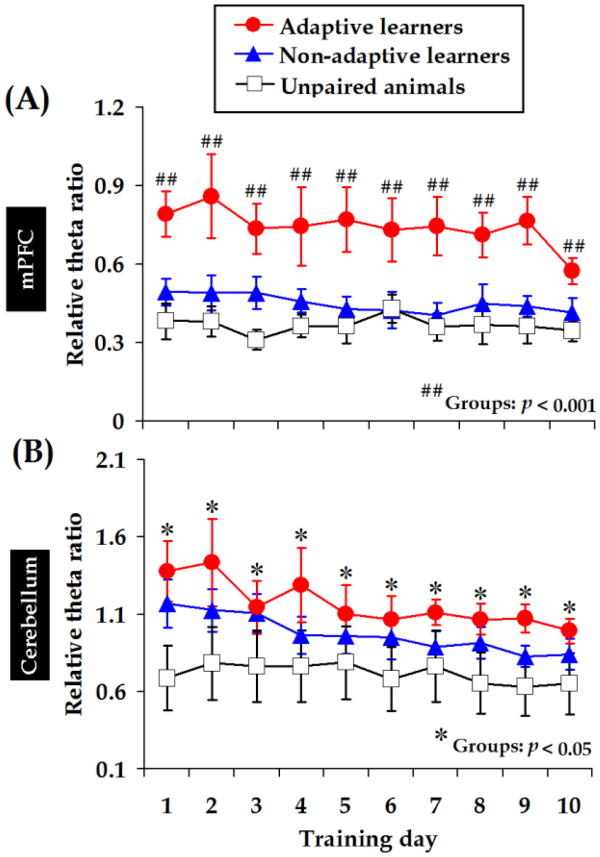

Figure S6 | Relative theta power in the mPFC and the cerebellum after the CS onset. (A) After the CS onset, relative theta power in the mPFC of adaptive learners (*n* = 11) remained relatively stable across ten training days ($F_{[9, 270]} = 1.534$, $p = 0.136$), but was stronger than in the non-adaptive learners (*n* = 12) and unpaired animals (*n* = 11) ($F_{[2, 31]} = 11.088$, $p < 0.001$, Group X Training Day interaction: $F_{[18, 279]} = 0.594$, $p = 0.903$, two-way ANOVA with repeated measures). **(B)** Likewise, cerebellar theta power in the adaptive learners was stronger than in the non-adaptive learners and the unpaired animals ($F_{[2, 31]} = 4.438$, $p = 0.020$, Group X Day of training interaction: $F_{[18, 279]} = 0.629$, $p = 0.876$, two-way ANOVA with repeated measures), although cerebellar theta power in all three groups of animals clearly decreased across ten training days ($F_{[9, 279]} = 2.958$, $p = 0.002$).

Figure_S7

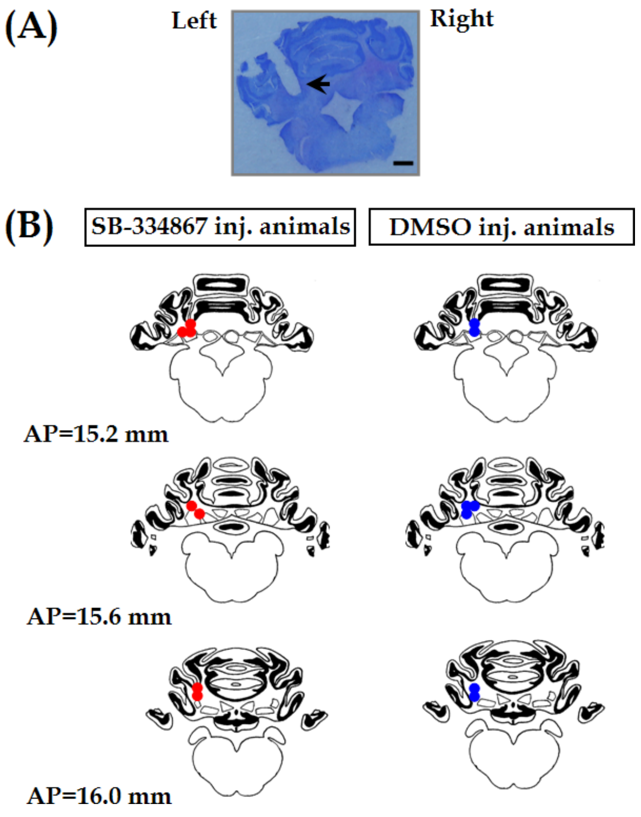

Figure S7 | Histological examination of the cannula tips in the cerebellum. (A) Representative toluidine blue-stained coronal cerebellar slice of a guinea pig deemed valid. A guide cannula (indicated by the black arrow) passed through the cerebellar cortex and its tip lay dorsal to the left intermediate cerebellum. Scale bar = 1.0 mm. **(B)** Line drawings of cerebellar coronal sections showing the location of drug injection sites (indicated by red circles for the SB-334867-injection animals and blue circles for the DMSO-injection animals, *n* = 7 each group) in the cerebellum. The black regions represent the granular cell layer (GCL) of cerebellar cortex. Numbers represent the distance (in mm) between the sections and Bregma.

Figure_S8

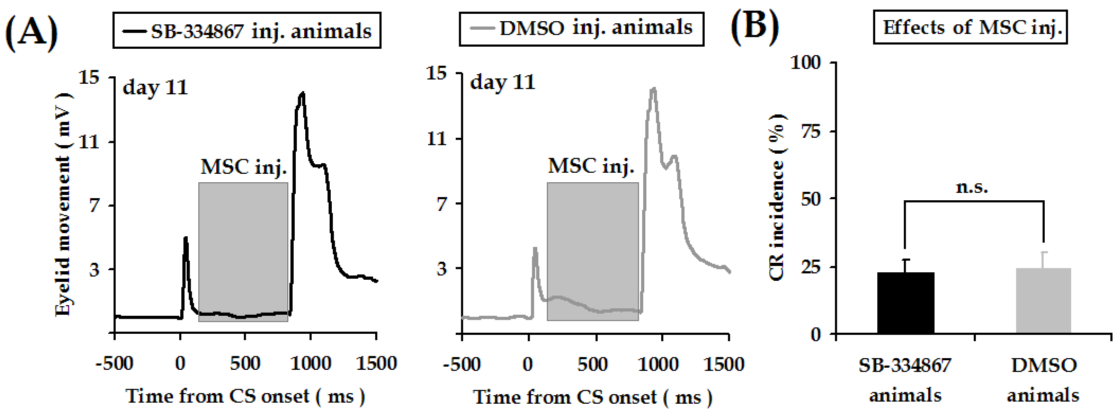

Figure S8 | Effects of microinjections of muscimol (MSC, 1.0 µg) into the intermediate cerebellum on the expression of acquired trace CRs on day 11. **(A)** The MSC injections severely impaired the expression of acquired trace CRs in both the SB-injection (*n* = 7) and the DMSO-injection (*n* = 7) groups of animals. **(B)** After the MSC injections, there was no significant difference in the CR incidence between SB-injection and DMSO-injection animals on day 11.
